# A new cow identification method using near-infrared spectral measurements and main components of raw milk features

**DOI:** 10.1371/journal.pone.0329499

**Published:** 2025-08-14

**Authors:** Tugba Aydemir

**Affiliations:** Faculty of Science, Department of Physics, Recep Tayyip Erdogan University, Rize, Türkiye; Yantai Institute of Technology, CHINA

## Abstract

Recent advances in cow identification have been instrumental in enhancing understanding of disease progression, optimizing vaccination strategies, improving production management, ensuring animal traceability, and facilitating ownership assignment. Cow identification and tracking involve the precise recognition of individual cows and their products through unique identifiers or markers. Traditional methods like computer vision, ear tags, branding, tattooing, microchips, and other electrical methods have been widely employed for cow identification and tracking over an extended period of time. However, these methods are prone to reliability issues caused by external factors such as physical damage, tag loss, weather-induced fading or damage, and the need for a software-based management system with RFID, which may not always be satisfactory for identifying cows. Merging near-infrared spectroscopy and routinely collected main components of raw milk (fat, protein, lactose, urea, and somatic cell count) with artificial intelligence offers a non-invasive, data-driven approach for cow identification, potentially increasing applicability in farm environments where such milk data are already part of routine monitoring. In this study, we presented an alternative approach to cow identification utilizing near-infrared spectral measurements alongside laboratory reference values for the main components of raw milk. In order to test our proposed method, we used a publicly available and newly released dataset of 1224 different measurements collected from 41 cows over a period of 8 weeks. Depending on the considered measurements and number of cows, the Naïve Bayes, Decision Tree, and Support Vector Machines classifiers achieved classification accuracy rates of between 69.23%−98.63%, 61.87%−100%, and 58.53%−97.26%, respectively. We believe that the proposed method has great potential to be an alternative way for cow identification applications.

## 1. Introduction

The evolution of cow identification systems has progressed from manual methods to automated processes aided by image processing. Traditional approaches like ear tagging, ear notching, RFID techniques, and electronic devices have historically served as means for individual cow identification in farming practices. However, these methods come with drawbacks, including potential losses, duplication issues, electronic device malfunctions, and susceptibility to fraud involving tag numbers. A primary drawback of employing RFID-based systems for animal identification lies in the need for significant efforts and labor to configure RFID modules into an effective identification and tracking system. Additionally, the expenses associated with procuring and replacing RFID tags, along with the operational costs of the identification system, must be carefully considered. Cow identification systems offer extensive benefits, impacting various stakeholders ranging from animal producers and consumers to the food industry. These systems play a crucial role in containing the spread of animal diseases by enabling efficient control of vaccinations and providing insights into disease patterns. Consequently, they help livestock producers mitigate losses caused by diseases, decrease governmental expenses associated with disease eradication efforts, minimize trade disruptions, and streamline ownership management processes.

Implementing cow identification through biometrics introduces a realm of new algorithmic developments and methodologies. Biometrics relies on identifying animals based on their physical, anatomical, or molecular characteristics. Computer vision-based techniques introduce a fresh application within the domain of animal multimodal biometrics, specifically enabling the recognition of cattle from images. The heightened precision in recognizing unique features promises a distinct identifier for cow identification. Biometric techniques in cow management will enhance automation systems, facilitate cow classification, and aid in dairy livestock monitoring, which necessitates thorough breed association tracking and monitoring for milking and health concerns. Furthermore, biometrics enable the tracking of cows to ascertain their natural habitats, administer vaccinations against various diseases, comprehend animal disease patterns, manage production, ensure traceability, and assign ownership accurately. To establish an effective cow identification system, efficient feature extraction and machine learning algorithms are imperative for accurate cattle classification. In a computer vision-based study, Wang et al. (2023) introduced a novel computer vision approach aimed at automatically discerning multi-object cattle rumination and quantifying both rumination duration and chew count for each individual cow [1]. Initially, the heads of the cattle within the video footage underwent tracking via a sophisticated multi-object tracking algorithm. Their algorithm seamlessly integrated the You Only Look Once algorithm with the kernelized correlation filter. Subsequently, images of each cow’s head were captured and stored at a standardized size, sequentially numbered for reference. Following it, a specialized rumination recognition algorithm was developed, utilizing parameters acquired through the frame difference method to accurately compute rumination duration and chew count. In order to validate the practicality and effectiveness of this methodology, extensive testing was conducted on multi-object cattle rumination videos, with the obtained outcomes systematically compared against those derived from human observation. Notably, experimental results showcased an average error rate of 5.902% in rumination duration estimation and 8.126% in chew count estimation. In another study, Wu et al. (2023) [2] introduced a method capable of monitoring the respiratory behavior of multiple cows efficiently. Initially, leveraging a dataset comprising 4,000 manually labeled images, the You Only Look at Coefficients model underwent fine-tuning to optimize its ability to recognize and segment multiple cows. Notably, it was observed that respiratory behavior during resting phases provided valuable insights into the health status of the cows. Subsequent stages involved identifying distinct resting states (lying and standing) of individual cows through a combination of convolutional neural networks and bidirectional long and short-term memory algorithms. Finally, custom detection algorithms tailored for lying and standing resting were deployed to enable respiratory behavior monitoring. Evaluation across 60 videos, each featuring diverse interference factors, demonstrated exceptional performance, with respiratory behavior monitoring accuracy exceeding 90.00% in 54 videos and achieving a perfect accuracy of 100.00% in 4 videos. In a different computer vision-based work, Sian et al. [3] investigated individual cattle identification within a single breed, employing texture feature extraction techniques such as Weber’s Local Descriptor and Local Binary Pattern. They fused these features and employed a Support Vector Machines (SVM) classification model, achieving an accuracy rate of 95.3%.

Apart from computer vision-based studies, some works focused on discriminating cattle by using muzzle point images. For example, Kumar et al. (2017) [4] introduced a novel hybrid texture feature extraction method aimed at recognizing and classifying individual cattle based on their muzzle point image patterns. The patterns contained abundant skin texture details and unique features like beads and ridge patterns. Following a similar path, Noviyanto and Arymurthy (2013) [5] introduced a matching refinement technique within the Scale Invariant Feature Transform (SIFT) descriptor method to enhance the recognition of cattle using a database of 160 muzzle print images. This technique involved computing matching scores for key points in the muzzle print images through the application of the refinement process in the SIFT approach. However, the performance of the matching refinement method was compared to that of the original SIFT approach, resulting in an equal error rate value of 0.0167. In a similar direction, Kumar et al. (2018a) [6] and Kumar et al. (2018b) [7] demonstrated superior accuracy in analyzing muzzle images compared to face images. Building upon this, Bello et al. (2020) [8] enhanced results using a dataset comprising 1000 images of cattle, focusing on coat patterns and muzzles. As highlighted by various authors in the study, the utilization of muzzle images as a biometric trait for identification purposes has consistently outperformed alternative methods, as supported by Shojaeipour et al. (2021) [9]. In another study, Kaur et al. (2022) [10] introduced a method for automatically detecting cattle by leveraging biometric characteristics, specifically focusing on the muzzle pattern extracted from digital images of the animals. Their system for recognizing muzzle patterns treats the muzzle area within an image as the main biometric feature for cattle identification. Consequently, the images contained unique features such as beads and ridges within the muzzle points and had a maximum recognition accuracy of 79.60% with a random forest classifier. In another study, Kusakunniran et al. (2020) [11] presented a methodology involving a Haar feature-based cascade classifier coupled with a watershed technique for extracting features from the muzzle of cattle faces. Feature extraction was conducted through a bag of histograms of oriented gradients and histograms of local binary patterns, followed by classification using an SVM with histogram intersection. The experiments yielded a notable accuracy of 95%. The studies have shown the successful integration of near-infrared (NIR) spectroscopy with machine learning techniques in various domains. For example, Solihin et al. (2024) demonstrated the effectiveness of stacked ensemble machine learning in calibrating NIR spectral data across multiple datasets, achieving classification accuracies up to 100% [12]. In food safety, Ting et al. (2020) used NIR combined with PCA and logistic regression to detect melamine adulteration in milk powder with 100% detection accuracy [13]. Similar non-destructive approaches were employed by Al-Sanabani et al. (2019) for mango quality assessment using handheld NIR devices, with R² values reaching 0.96 using SVM regression [14]. In another study, Tan et al. (2021) applied NIR-based PCA and logistic regression to detect adulteration in stingless bee honey, achieving over 98% classification accuracy (CA) [15]. These examples highlight the growing potential and versatility of NIR-ML systems in both qualitative and quantitative assessment tasks. Inspired by these successes, our study explores the feasibility of using NIR spectral data and milk composition features, paired with machine learning classifiers, as a novel approach for cow identification.

While computer vision and muzzle image-based methods offer valuable solutions for cow identification, they also present several limitations. These include dependency on high-quality image acquisition (e.g., lighting, angle, resolution), significant variability in cow appearance (e.g., breed, coat color), and the complexity and cost of hardware and infrastructure. Moreover, such systems may struggle in outdoor or crowded environments and often require ongoing maintenance and calibration. Additionally, concerns related to data privacy and potential algorithmic bias may limit their adoption in certain settings.

In this study, we proposed an alternative cow identification solution that used NIR spectral measurements of raw milk samples collected over 8 weeks and laboratory reference values of milk, including fat, protein, lactose, urea, and somatic cell count (SCC). The Neighborhood Component Analysis (NCA) algorithm was applied to select the most informative features, which provided the highest CA performance in the training set. Afterward, the test set was classified by Naïve Bayes (NB), Decision Tree (DT), and SVM algorithms. The obtained results showed that the proposed method can be used as an alternative method for cow identification.

## 2. Materials and methods

In the following subsections, the dataset used is described. Afterward, the parts of the proposed method are introduced in detail*.*

### 2.1. Data set description

The dataset comprises 1224 NIR spectral measurements of raw milk samples and the main components of raw milk, including fat, protein, lactose, urea, and SCC. These measurements were collected over 8 weeks at the experimental farm ‘Hooibeekhoeve’ in Geel, Belgium, using a 256-pixel cooled InGaAs diode array NIR spectrometer (1.7−256 Plane Grating Spectrometer, Carl Zeiss, Jena, Germany). These measurements are in transmittance mode within the wavelength range of 960–1690 nm, with a resolution of 2.86 nm/pixel and a total of 256 wavelengths. Each spectral measurement includes readings of the raw milk sample, a white spectral reference, and a dark spectral reference, all with an integration time of 100 ms and averaged over 100 measurements. Laboratory reference values are available for the main components of raw milk including fat, protein, lactose, urea, and SCC. The dataset was collected from 41 cows using a VMS^TM^ (DeLaval, Sweden) automatic milking system. The aim is to categorize the cows in the testing set into the correct labels. The cows, at an average lactation stage of 168 ± 84 days in milk and parity of 2.0 ± 1.1, were milked 2.6 times daily on average. However, spectral analysis was only carried out when a sample was captured for reference analysis as part of an unrelated feeding trial. The total operation time of milk sample collection ranged from 21 to 85 hours per week, with an average of 158 samples collected each week. The raw milk samples collected for laboratory analysis were treated with 0.3 mg/mL of bronopol and underwent analysis at the Milk Control Center (MCC Vlaanderen, Lier, Belgium) within no more than three days following sample collection. The laboratory reference values for fat, protein, lactose, and urea for each sample were determined under ISO 9622 and ISO 13366−2 for SCC [16].

### 2.2. Preprocessing, feature selection and classification procedures

The provided spectral data was collected in different sessions with about one week in between. This is an important point to pay attention to. Because air conditions, dietary changes, and other environmental situations might influence the content of the cows’ milk in different sessions. Therefore, the changes in milk content would naturally affect the measured spectral data. This session-to-session variation can directly influence the classification performance of the test set. Hence, a normalization process should be implemented in the training set and the test set to alleviate the impact of the magnitude change. In this study, a variance normalization process was implemented for all trials [17]. All spectral data trials were normalized to each trial’s standard deviation as given in Equation 1, where *S, S*_*N*_, and *std* represent a spectral data trial, the normalized version of *S*, and the standard deviation operator, respectively.


$$S_{N}=\frac{S}{std(S)}$$ (1)

The estimated standard deviation of a spectral data trial was calculated as follows:


$$std(S)=\sqrt{\frac{\sum {\left(S-\overset{―}{S}\right)}^{2}}{L-1}}$$ (2)

where $\overset{―}{S}$ is the mean value of all spectral data values of the trial and *L* is the length of the trials, which was equal to 256 in this study. An example of a raw signal and its normalized state are shown in Fig 2. Although only variance normalization was applied in this study, future work will explore the application of scatter correction techniques such as Standard Normal Variate, Multiplicative Scatter Correction, and Extended Multiplicative Signal Correction to further improve spectral data quality and classification performance.

**Fig 1:**
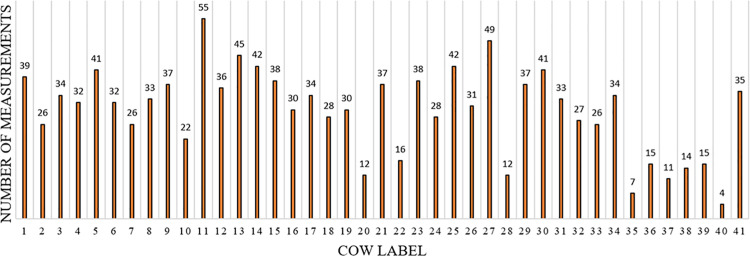
Number of measurements for each cow.

In this study, we used the 256 spectral data values and the main components of raw milk, including fat rate, protein rate, lactose rate, urea rate, and SCC, as features. Table 1 shows the order of features, which will be meaningful in the results section and will be represented by their index number as 1 to 261. It is very well known from the literature that excluding redundant features and selecting the most informative ones enhances the classification accuracy of classifiers [18–20]. To do this, we utilized neighborhood component analysis, which is an effective method for feature selection in pattern recognition applications on the training dataset [21–23]. It is worth noting that the training and test datasets were formed by randomly shuffling and splitting the whole dataset into two equal parts, and a leave-one-out-cross-validation (LOOCV) procedure was applied for the training stage. Fig 3 shows the flowchart of the proposed method [24].

**Table 1:** The order of the features.

Feature 1	Feature 2	Feature 3	Feature 4	Feature 5	Feature 6 to 261
Fat rate	Protein rate	Lactose rate	Urea rate	SCC	256 spectral data measurements in increasing order

**Fig 2:**
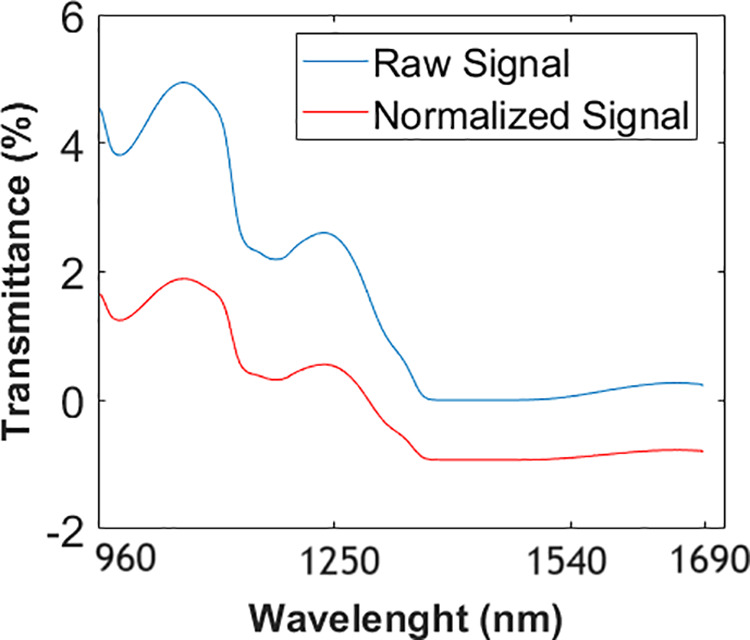
Raw and normalized signals.

**Fig 3:**
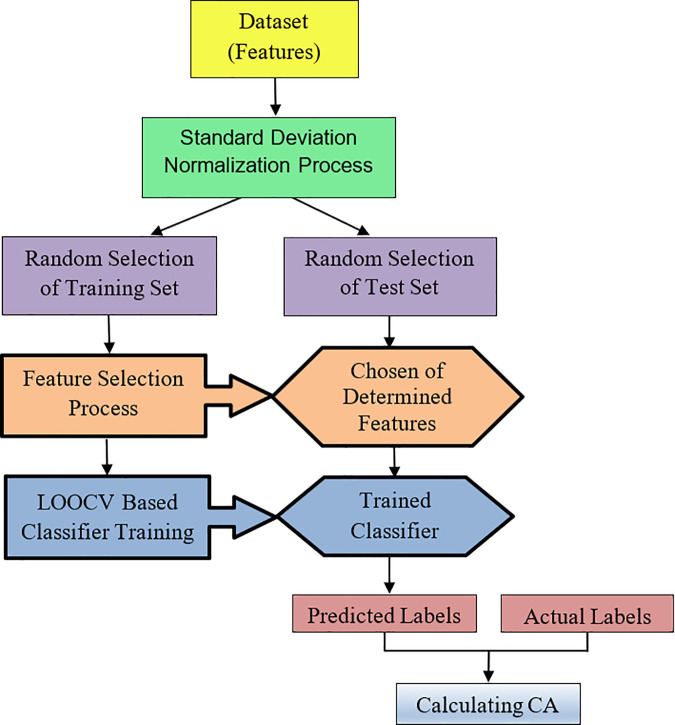
The flowchart of the proposed method.

The process of feature selection involves identifying the optimal subset of features, denoted as ‘*m*’, from a pool of ‘*N*’ features within the training dataset. This selection relies on evaluating criteria established by the chosen algorithm. Despite its benefits, such as dimensionality reduction and algorithm performance enhancement, there’s no assurance that the selected feature combination will yield the highest CA. In our study, alongside the main components of raw milk features, we directly incorporated transmittance spectra values from spectroscopic data as features. We selected the most suitable transmittance spectra values of wavelengths and the main components of raw milk, which provided higher CA performance in the training set. To achieve this, we employed the NCA method for feature selection. The test CA was calculated as the percentage of correctly classified samples over the total number of samples in the test set.

NCA operates as a method for weighting features based on their relevance, prioritizing them in descending order of significance [25,26]. Its core objective lies in deriving a weighting vector, ‘*w*’, which facilitates the selection of a feature subset ideal for nearest neighbor classification optimization. The weighted distance, denoted as *D*_*w*_, between two features, *f*_*i*_ and *f*_*j*_, is mathematically computed as follows:


$$D_{w}\left(f_{i},f_{j}\right)=\sum_{n=1}^{m}{w_{n}}^{2}\left|f_{i,n}-f_{j,n}\right|$$ (3)

where *w*_*n*_ represents the weight associated with the *n*^*th*^ feature. The association between the weighted distance *D*_*w*_ and the probability *P*_*ij*_ can be mathematically calculated as follows:


$$P_{ij}=\left\{ \begin{array}{cc} \frac{k\left(D_{w}(f_{i},f_{j})\right)}{\sum_{j=1}^{L}k\left(D_{w}(f_{i},f_{j})\right)} & if\;i\neq j\\ 0 & if\;i=j \end{array}\right.\;$$ (4)

where $k\left(D_{w}(f_{i},f_{j})\right)$ is a kernel function defining the similarity between *f*_*i*_ and *f*_*j*_. Furthermore, by assigning *V*_*b*_ to be the output value calculated by a randomized regression model, *V*_*a*_ to be the true value for *fi*, and *C* to be the loss function that measures the difference between *Va* and *Vb*, the average value of *C(Vb,Va)* becomes


$$C_{i}=E\left(C\left(V_{b},V_{a}\right)\right)=\sum_{j=1}^{L}p_{ij}(V_{b},V_{a})$$ (5)

where j≠i. The loss function $C\left(V_{b},V_{a}\right)$ used for this study is the mean squared prediction error, which is equal to $e_{ij}={\left(V_{j}-V_{i}\right)}^{2}$. The probability of the correct classification of *f*_*i*_ can be calculated as shown in Equation 6.


$$P_{i}=\left\{ \begin{array}{cc} \sum_{j=1}^{L}p_{ij}e_{ij} & if\;i=j\\ 0 & if\;i\neq j\; \end{array}\right.\;$$ (6)

The goal of minimizing prediction error in neighborhood component analysis is achieved through the computation outlined in Equation 7, which represents the objective function.


$$O(w)=\frac{1}{L}\sum_{i=1}^{L}P_{i}+\alpha \sum_{b=1}^{B}{w_{b}}^{2}\mathrm{\backslash ]}$$ (7)

where *w*_*b*_ is the *b*^*th*^ element in vector *w* and *α* represents the regularization parameter, which enables overcoming the overfitting problem, and its optimal value was determined in the LOOCV stage, which used the training set. After determining the most discriminative features by applying the NCA algorithm to the training set, we kept the selected features and excluded the rest of them from the test set for the classification stage. As classifiers, we used the most recommended algorithms, including the NB, the DT, and the SVM, which are briefly introduced below.

NB assumes that the presence of a particular feature in a class is unrelated to the presence of any other feature. It calculates the probability of each class given a set of features and selects the class with the highest probability, as given in Equation 8.


$$P\left(y\backslash x_{1},x_{2},\ldots ,x_{n}\right)=\frac{P\left(x_{1},x_{2},\ldots ,x_{n}\backslash y\right)xP(y)}{P\left(x_{1},x_{2},\ldots ,x_{n}\right)}$$ (8)

where $P\left(y\backslash x_{1},x_{2},\ldots ,x_{n}\right)$ is the posterior probability of class *y* given features *x*_*1*_*, x*_*2*_*,..,x*_*n*_, $P\left(x_{1},x_{2},\ldots ,x_{n}\backslash y\right)$ is the likelihood of observing features x_1_, x_2_,..,x_n_ given class *y*, *P(y)* is the prior probability of class *y*, $P\left(x_{1},x_{2},\ldots ,x_{n}\right)$ is the probability of observing features *x*_*1*_*, x*_*2*_*,..,x*_*n*_. NB is preferred for its speed and simplicity, which make it suitable for large datasets with high dimensions. The classification decision is made by selecting the class that class maximizes the posterior probability.

We also tested the proposed method with a DT classifier, which recursively splits the feature space into partitions based on feature values. At each internal node of the tree, a decision is made according to the value of a particular feature. Mathematically, it can be represented as a series of *if-else* conditions. The split at each node is chosen to maximize the information gain or minimize impurities in the resulting partitions. In this study, Gini impurity and entropy were used to measure the impurity. We continued this process until a stopping criterion was met when reaching a maximum tree depth or minimum number of samples in a node.

As a third classifier, we utilized SVM, which is one of the most commonly employed algorithms in pattern recognition and machine learning communities. It aims to find the hyperplane that best separates the data into classes while maximizing the margin between them. It particularly works well in high-dimensional spaces and is effective even in cases where the number of dimensions exceeds the number of samples. Mathematically, given a training dataset ${\left\{(g_{i},l_{i})\right\}}_{i=1}^{n}$, where *g*_*i*_ are the feature vectors and *l*_*i*_ are class *l*abels (+1 or −1), SVM seeks to solve the optimization problem $\min_{w,b}\frac{1}{2}{\left\|w\right\|}^{2}$ subject to $l_{i}\left(w.g_{i}+b\right)\geq 1\;$for *i = 1, 2, …, n*. Here, w is the weight vector, *b* is the bias term, and ‖.‖ denotes the Euclidean norm. SVM can be extended to handle non-linearly separable data usin*g* the kernel trick, where the input space is mapped into a higher-dimensional space where the data becomes linearly separable. In this study, for the SVM classifier, the most commonly used radial basis function (RBF) kernel was utilized to train the SVM, and the RBF kernel was used with MATLAB’s default settings: BoxConstraint (C) = 1 and KernelScale (γ) = ‘auto’, allowing automatic scaling based on the training set.

## 3. Results

**Fig 4:**
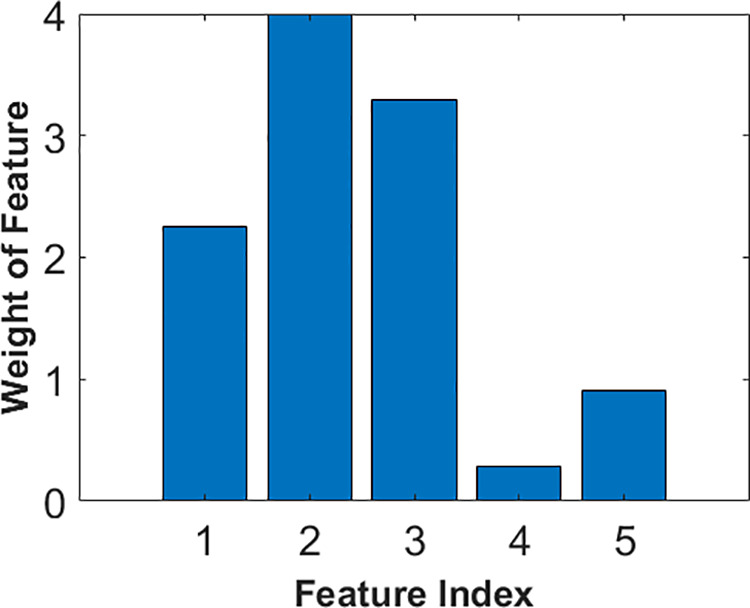
The weights of feature indexes 1 through 5.

**Fig 5:**
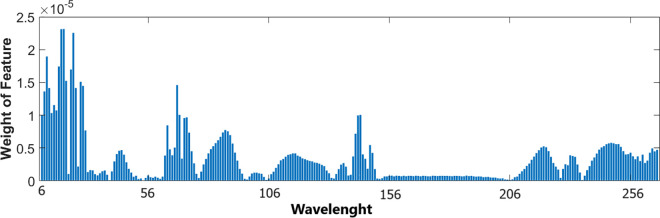
The weights of feature indexes 6 through 261.

In this study, we weighted the features by using the NCA and tested the proposed method by applying the widely used classification algorithms, including NB, DT, and SVM, to selected features. Figs 4 and 5 show the weight of the main components of raw milk (fat rate, protein rate, lactose rate, urea rate, and SCC) features and spectral data features (feature index of 6 to 261), respectively. It is clear from Fig 4 that while the protein rate is the most informative feature, the urea rate is seen as the least discriminative feature among the other main components of raw milk. According to the weight of spectral data features, it is understood that low-spectral measurements are more informative than the rest of the measurements because shorter wavelengths interact more strongly with molecular structures, leading to distinct transmission variations. As the wavelength decreases, light penetration is reduced, making the transmitted signal more sensitive to subtle compositional differences in the sample. This enhanced sensitivity improves spectral resolution, allowing for clearer differentiation of chemical components based on their transmittance properties.

In order to see the effectiveness of the main components of raw milk and spectral data features, we tested them individually and also together (hybrid usage of features). The proposed method was tested by the sub-datasets, which were created according to the considered number of measurements (NoM) from a cow. For example, if the NoM parameter was 45, the sub-dataset included the measurements recorded from the cows numbered as 11, 13 and 27 as shown Fig 1. Afterward, this 3-class identification problem was performed as represented in Fig 3. Table 2 shows the best CA results of the main components of raw milk features for each NoM. In this table NoM and Number of Animals (NoA) are given in the first two columns, respectively. In the last three columns, the highest CA results are provided according to the selected features. It should be noted that the numbers of selected features (NoSF) are shown in parentheses beside each CA. It is worthwhile mentioning that the NCA evaluates all the features together and ranks them from the most effective to the least effective according to their weights. Classifiers use the most effective features that provide the highest classification accuracy among these ranked features. It is naturally understandable from the table that as the NoM value increases (or NoA decreases), CA performance generally increases. In this table, the overall best CA was achieved by NB as 97.26% when NoM was 45. For the same NoM value, DT and SVM classifiers obtained their highest values as 94.52% and 90.41%, respectively. However, it should be noted that for this condition, the number of cows was only 3. Nevertheless, for the higher values of NoA, the obtained results showed notable performance for the cow identification process. The results also showed that all five components of raw milk features were generally selected by the NCA algorithm as informative. The best CA results of the spectral data features for each NoM were presented by Table 3. Compared to the main components of raw milk features, the spectral data features significantly obtained lower CA performances that the highest CA values for NB, DT and SVM were achieved as 61.64%, 68.49% and 50.69%, respectively. Another important point was that the SVM classifier used the most NoSF for every NoM value. To validate the reported classification accuracy results, a cross-validation approach was used, and all classifiers were evaluated using the same test conditions. The confidence intervals for classification accuracies were computed to account for potential variations in performance across randomly split datasets.

**Table 2:** The best CA results of the main components of raw milk features for each NoM.

NoM	NoA	NB – NoSF	DT- NoSF	SVM – NoSF
10	39	60.70 (5)	50.17 (5)	52.51 (5)
15	35	61.85 (5)	50.52 (5)	55.92 (5)
20	32	64.13 (5)	54.71 (5)	54.71 (5)
25	31	66.54 (5)	54.34 (5)	58.41 (5)
30	25	69.41 (5)	60.52 (5)	60.30 (5)
35	15	70.43 (5)	63.78 (5)	66.45 (5)
40	7	83.94 (4)	77.42 (4)	76.77 (4)
45	3	97.26 (4)	94.52 (4)	90.41 (3)

**Table 3:** The best CA results of the spectral data features for each NoM.

NoM	NoA	NB – NoSF	DT- NoSF	SVM – NoSF
10	39	26.42 (20)	29.26 (206)	14.05 (208)
15	35	26.65 (137)	30.14 (128)	14.24 (254)
20	32	28.08 (10)	31.34 (86)	15.94 (234)
25	31	29.39 (19)	31.05 (83)	15.71 (234)
30	25	31.61 (71)	33.27 (117)	16.92 (236)
35	15	40.53 (13)	44.52 (203)	22.92 (226)
40	7	53.55 (50)	57.42 (164)	35.48 (196)
45	3	61.64 (144)	68.49 (144)	50.69 (253)

**Fig 6:**
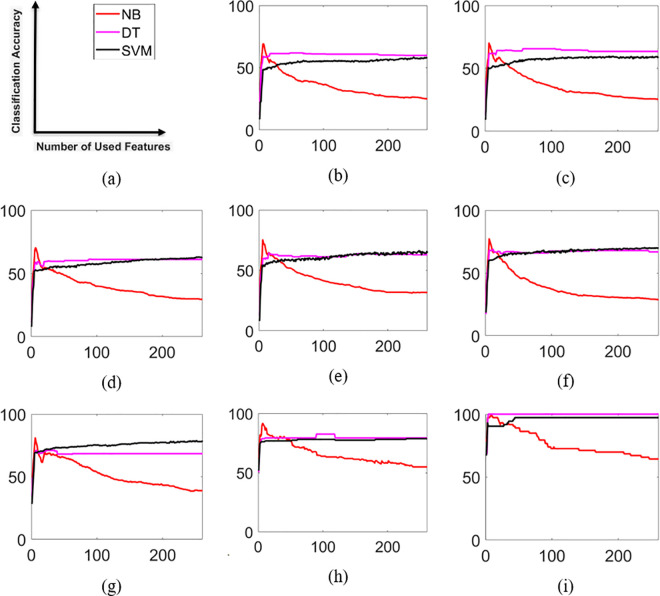
The entire results of classifiers according to the weight of features; (a) the legend and axis names of the figures, (b) the results for NoM  = 10 and NoA = 39, (c) the results for NoM = 15 and NoA = 35, (d) the results for NoM = 20 and NoA = 32, (e) the results for NoM = 25 and NoA = 31, (f) the results for NoM = 30 and NoA = 25, (g) the results for NoM = 35 and NoA = 15, (h) the results for NoM = 40 and NoA = 7, (i) the results for NoM = 45 and NoA = 3.

Fig 6 shows the entire results of classifiers according to the weight of whole features (the main components of raw milk features and the spectral data features). It is clearly understood from this figure that the NB classifier got the highest CA performance for the small number of features. Because it assumes feature independence, making it less susceptible to the curse of dimensionality. Unlike other classifiers, NB does not rely on complex decision boundaries, which allows it to maintain stable performance even with limited features.

On the other hand, its performance decreased dramatically as the number of used features increased because the low-weight features included in the classification stage have less discriminative power and act as redundant features that carry common information between classes. On the other hand, while the general performances of the DT and SVM classifiers were close to each other, the DT had slightly better CA performance than the SVM classifier. The best CA results of classifiers for each NoMs are presented in Table 4 in terms of percentage. In this table, NoMs and NoAs are given in the first two columns, respectively. In the last three columns the highest CA results are provided. It should be noted that beside each CA NoSFs are shown in the parenthesis. It is naturally understandable from the table that as NoM increases (or NoA decreases), CA also increases, and the lowest performances are calculated for the highest NoM. Compared with the DT and the SVM, the NB classifier achieved the highest CA performances for every NoM value except 45. It provided between 69.23% and 98.63% classification accuracy performances by using 6 or 7 selected features. DT was the only classifier that achieved 100% CA performance using only 4 selected features. It obtained the lowest CA performance as 61.87% when NoM was 10. The DT classifier achieved the highest CA values using between 4 and 90 selected features. One of the remarkable results was obtained with the SVM classifier, which used over 229 NoSF except for 15 and 45 NoM values. The lowest performances were generally calculated with the SVM. While it obtained the poorest CA performance as 58.53% when NoM was 10 by using 240 selected features, the highest CA rate was calculated as 97.26% when NoM was 45.

**Table 4:** The best CA results of hybrid usage of features for each NoM.

NoM	NoA	NB – NoSF	DT- NoSF	SVM – NoSF
10	39	69.23 (7)	61.87 (48)	58.53 (240)
15	35	70.21 (6)	62.68 (57)	59.76 (187)
20	32	70.29 (7)	63.23 (88)	63.04 (252)
25	31	75.23 (6)	64.33 (15)	66.36 (248)
30	25	77.22 (6)	68.98 (12)	70.28 (240)
35	15	81.06 (6)	71.10 (8)	78.74 (242)
40	7	91.61 (7)	82.58 (90)	79.71 (229)
45	3	98.63 (7)	100 (14)	97.26 (44)

To validate the reported classification accuracy results, a cross-validation approach was used, and all classifiers were evaluated using the same test conditions. The confidence intervals for classification accuracies were computed to account for potential variations in performance across randomly split datasets. The results demonstrate that the NB classifier consistently achieved higher classification accuracy with a small number of selected features, whereas the DT classifier provided perfect classification in a limited subset of cases. The classification experiments were conducted using MATLAB R2023b with the Statistics and Machine Learning Toolbox. The computational environment consisted of a 12^th^ Gen Intel® Core™ i7-1260P processor (2.10 GHz, x64 architecture), running on a 64-bit operating system. The experiments were performed on a standard laptop computer without the use of GPU acceleration.

## 4. Conclusion

In this study, the proposed solution has great potential to identify cows from the spectral data and the main components of raw milk, including fat rate, protein rate, lactose rate, urea rate, and SCC. This study also plays an essential role in preventing social, electronic, and physical problems that can be caused by conventional identification approaches. Such a system makes recognition tasks easier and faster. The proposed method is developed as an alternative cow identification model to the existing solutions. To the best of our knowledge, this is the first study that uses spectral data and the main components of raw milk as features for identifying cows. While the study does not include a direct performance comparison with existing identification technologies such as RFID or biometric systems, the proposed method introduces a novel, non-invasive approach that leverages routinely collected milk data, potentially offering a complementary or alternative solution for cow identification.

In order to train and test the proposed cow recognition method, we have used a publicly available and newly released dataset of 1224 different measurements collected from 41 cows. These measurements were considered as features and the most informative ones were selected by the NCA algorithm. Individually used features observed that the main components of raw milk features provided better CA performance than the spectral data features. On the other hand, the results of hybrid usage of features showed that while the DT classifier achieved 100% CA performance for only 3-cow identification, the NB classifier has great potential to recognize a cow population larger than 3. Moreover, it should be noted that the NB classifier required only 6 or 7 selected features.

The proposed method shows great promise as an alternative approach for cow identification using near-infrared spectral measurements and the main components of raw milk. While the results indicate high classification accuracy, it is essential to acknowledge that the performance of machine learning-based classification models is influenced by dataset size, feature selection methods, and classifier choice. Although our approach demonstrated 100% accuracy in a subset of cases, it should be noted that performance may vary under different environmental conditions or when applied to a larger dataset with more diverse cow populations. Future research should focus on validating the approach across different farms, breeds, and environmental settings. The study was conducted using data from 41 cows over an 8-week period. While this dataset provides a solid foundation, a larger dataset with diverse breeds and varying environmental conditions is necessary to fully evaluate the generalizability of the model. Nevertheless, it is thought that this study is valuable as it demonstrates that cow identification can be performed using milk composition and near-infrared spectral measurements. On the other hand, the study did not include a direct performance comparison with traditional cow identification methods such as RFID or biometric imaging techniques. Future research should evaluate the cost-effectiveness and feasibility of integrating this method into real-world farm management systems. Moreover, we will investigate the use of advanced ensemble learning methods such as Random Forest and XGBoost to further improve classification accuracy and model robustness

## References

[1] Wang Y, Chen T, Li B, Li Q. Automatic identification and analysis of multi-object cattle rumination based on computer vision. J Anim Sci Technol. 2023;65(3):519–34. doi: 10.5187/jast.2022.e87 37332285 PMC10271932

[2] Wu D, Han M, Song H, Song L, Duan Y. Monitoring the respiratory behavior of multiple cows based on computer vision and deep learning. J Dairy Sci. 2023;106(4):2963–79. doi: 10.3168/jds.2022-22501 36797189

[3] Sian C, Jiye W, Ru Z, Lizhi Z. Cattle identification using muzzle print images based on feature fusion. IOP Conf Ser: Mater Sci Eng. 2020;853(1):012051. doi: 10.1088/1757-899x/853/1/012051

[4] Kumar S, Singh SK. Automatic identification of cattle using muzzle point pattern: a hybrid feature extraction and classification paradigm. Multimedia Tools and Applications. 2017;76:26551–80.

[5] Noviyanto A, Arymurthy AM. Beef cattle identification based on muzzle pattern using a matching refinement technique in the SIFT method. Computers and electronics in agriculture. 2013;99:77–84.

[6] Kumar S, Pandey A, Satwik KSR, Kumar S, Singh SK, Singh AK, et al. Deep learning framework for identification of cattle using muzzle point image pattern. Measurement. 2018a;116:1–17.

[7] Kumar S, Singh SK, Abidi AI, Datta D, Sangaiah AK. Group sparse representation approach for identification of cattle on muzzle point images. Int J Parallel Prog. 2018b;46(5):812–37.

[8] Bello R, Talib A, Mohamed A. Deep learning-based architectures for recognition of cow using cow nose image pattern. Gazi Uni J Sci. 2020;1(1):1.

[9] Shojaeipour A, Falzon G, Kwan P, Hadavi N, Cowley FC, Paul D. Automated muzzle detection and biometric identification via few-shot deep transfer learning of mixed breed cattle. Agronomy. 2021;11(11):2365.

[10] Kaur A, Kumar M, Jindal MK. Shi-Tomasi corner detector for cattle identification from muzzle print image pattern. Ecological Informatics. 2022;68:101549.

[11] Kusakunniran W, Wiratsudakul A, Chuachan U, Kanchanapreechakorn S, Imaromkul T, Suksriupatham N, et al. Biometric for cattle identification using muzzle patterns. International Journal of Pattern Recognition and Artificial Intelligence. 2020;34(12):2056007.

[12] Solihin MI, Yuan CJ, Hong WS, Pui LP, Kit AC, Hossain W, et al. Spectroscopy data calibration using stacked ensemble machine learning. IIUM Engineering Journal. 2024;25(1):208–24.

[13] Ting DF, Pui LP, Solihin MI. Feasibility of fraud detection in milk powder using a handheld near-infrared spectroscopy. In AIP Conference Proceedings. 2020. p. 1.

[14] Al-Sanabani A, D. G. DG, Solihin MI, Pui LP, Astuti W, Ang CK, et al. Development of non-destructive mango assessment using handheld spectroscopy and machine learning regression. In: Journal of Physics: Conference Series. 2019. p. 012030.

[15] Tan SH, Pui LP, Solihin MI, Keat KS, Lim WH, Ang CK. Physicochemical analysis and adulteration detection in Malaysia stingless bee honey using a handheld near‐infrared spectrometer. Journal of Food Processing and Preservation. 2021;45(7):e15576.

[16] Diaz-Olivares JA, van Nuenen A, Gote MJ, Díaz VF, Saeys W, Adriaens I, et al. Near-infrared spectra dataset of milk composition in transmittance mode. Data Brief. 2023;51:109767. doi: 10.1016/j.dib.2023.109767 38075623 PMC10700509

[17] Aydemir O, Kayikcioglu T. Wavelet transform based classification of invasive brain computer interface data. Radioengineering. 2011;20(1):31–8.

[18] Aydemir T, Şahin M, Aydemir O. Sequential forward mother wavelet selection method for mental workload assessment on N-back task using photoplethysmography signals. Infrared Physics & Technology. 2021;119:103966.

[19] Ergün E. Deep learning based multiclass classification for citrus anomaly detection in agriculture. Signal, Image and Video Processing. 2024;18(11):8077–88.

[20] Aydemir O. Prediction of six products from the cucurbitaceae family using visible/near-infrared spectroscopic data. Journal of Testing and Evaluation. 2023;51(2):979–88.

[21] Akinpelu S, Viriri S. Speech emotion classification using attention based network and regularized feature selection. Sci Rep. 2023;13(1):11990. doi: 10.1038/s41598-023-38868-2 37491423 PMC10368662

[22] Aydemir Ö. Combining sub-band power features extracted from different time segments of EEG trials. In: IEEE 2017 40th International Conference on Telecommunications and Signal Processing (TSP). 2017. p. 383–6.

[23] Chen W, Cai Y, Li A, Su Y, Jiang K. EEG feature selection method based on maximum information coefficient and quantum particle swarm. Sci Rep. 2023;13(1):14515. doi: 10.1038/s41598-023-41682-5 37666919 PMC10477332

[24] Aydemir T, Şahin M, Aydemir O. A new method for activity monitoring using photoplethysmography signals recorded by wireless sensor. Journal of Medical and Biological Engineering. 2020;40(6):934–42.

[25] Lan T, Tong C, Chen X, Shi X, Chen Y. KPI relevant and irrelevant fault monitoring with neighborhood component analysis and two-level PLS. Journal of the Franklin Institute. 2018;355(16):8049–64.

[26] Rajevenceltha J, Gaidhane VH. A no-reference image quality assessment model based on neighborhood component analysis and Gaussian process. Journal of Visual Communication and Image Representation. 2024;98:104041.

